# Fluorescent Reporter Libraries as Useful Tools for Optimizing Microbial Cell Factories: A Review of the Current Methods and Applications

**DOI:** 10.3389/fbioe.2015.00147

**Published:** 2015-09-28

**Authors:** Frank Delvigne, Hélène Pêcheux, Cédric Tarayre

**Affiliations:** ^1^Microbial Processes and Interactions (MiPI), Gembloux Agro-Bio Tech, University of Liège, Gembloux, Belgium

**Keywords:** mini-bioreactors, high-throughput, flow cytometry, single cell

## Abstract

The use of genetically encoded fluorescent reporters allows speeding up the initial optimization steps of microbial bioprocesses. These reporters can be used for determining the expression level of a particular promoter, not only the synthesis of a specific protein but also the content of intracellular metabolites. The level of protein/metabolite is thus proportional to a fluorescence signal. By this way, mean expression profiles of protein/metabolites can be determined non-invasively at a high-throughput rate, allowing the rapid identification of the best producers. Actually, different kinds of reporter systems are available, as well as specific cultivation devices allowing the on-line recording of the fluorescent signal. Cell-to-cell variability is another important phenomenon that can be integrated into the screening procedures for the selection of more efficient microbial cell factories.

## Fluorescent Proteins as Reporter: From the Single-Cell Challenge to the Advent of Synthetic Biology

**Figure 1:**
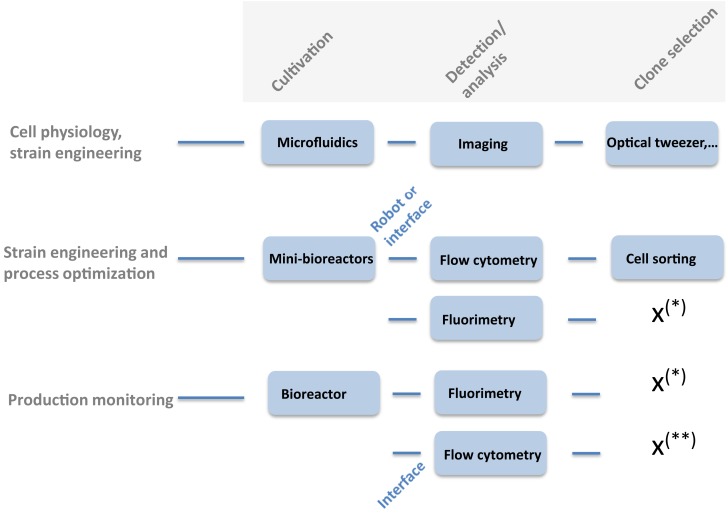
**Diversity of experimental devices that can be used for investigating fluorescent reporter libraries**. (*) Device not compatible with cell sorting. (**) Cell sorting is not considered in bioprocessing conditions.

Besides its importance in giving new insights in cell biology, e.g., for the analysis of the intrinsic and extrinsic component of phenotypic noise among microbial population (Swain et al., 2002), fluorescent reporters are also an important component for the development of new bioprocesses, i.e., for strain engineering and process optimization up to the large-scale production of bioproducts (Polizzi and Kontoravdi, 2014). From a fundamental perspective, fluorescent reporter libraries are available for several model organisms, including *Escherichia coli* K12 MG1655 (Zaslaver et al., 2006) and *Saccharomyces cerevisiae* (Newman et al., 2006). The two above-mentioned fluorescent reporter libraries have notably been used for the characterization of noise in protein expression (Newman et al., 2006; Silander et al., 2012). Indeed, molecular processes associated with DNA transcription and translation are subjected to different noise mechanisms leading a cell-to-cell variability in protein content among an isogenic microbial population (Sanchez et al., 2013). Clone libraries and experimental devices for the cultivation and the detection of fluorescent signal at the single-cell level have been specifically developed (Taniguchi et al., 2010). Besides these genome-scale libraries, fluorescent reporter system can also be used for the design of smaller libraries, e.g., for the estimation of the strength of several promoters that could be used for the expression of a protein of interest or for the design of synthetic metabolic pathways (Xu et al., 2012). This last application of fluorescent reporter is very important, since synthetic biology becomes widespread for the design of efficient cell factories, able to synthesize fuels and chemicals with high titer. Fluorescent proteins can also be found in more specific applications, such as the detection of the intracellular metabolite level (Schallmey et al., 2014) or the control of lab evolution (Reyes, 2012a,b), which will be detailed throughout this review. The exploitation of a fluorescent reporter library is greatly facilitated by the use of specific experimental devices. Indeed, the actual experimental toolbox dedicated to the use of fluorescent reporters allows for all the manipulations required in bioprocess optimization and scale-up and comprise specific cultivation devices, analytical tools, and clone selection tools (Figure 1). Among the cultivation device, a full range of culture volume is available, from micro- (picoliter) and mini-bioreactor (milliliter) to full-scale bioreactors (liter). Micro-bioreactors are based on microfluidic chips adapted to the culture of microorganisms. A nice example of micro-bioreactor has been developed by Grunberger et al. (2012, 2013), where a single microbial cell is isolated in a picoliter chamber perfused by fresh medium. The height of the picoliter chamber is designed in order to be slightly higher than the mean diameter of the microbial cells, so that microbial cells are maintained in the chamber and are continually fed with fresh medium whereas metabolites and by-products are continuously extracted. The perfusion mode of culture allows thus to cultivate microorganisms under constant environmental conditions. Imaging allows for the acquisition of the individual division rate and also the gene activity if linked with a fluorescent reporter system. A major limitation of the actual micro-bioreactor is that they are not designed to work in the operating modes generally met in industrial conditions, i.e., batch and fed-batch (Love, 2013; Grunberger et al., 2014). This limitation can be overcome by considering mini-bioreactors. This range of bioreactor involves the use of cultivation volume of around 1 ml (Klockner and Buchs, 2012). One of the most advanced mini-bioreactor platform to date is the Biolector system, and its extension Robolector (Funke et al., 2010). This device is based on a microplate and allows the parallel cultivation of 48 samples with on-line determination of biomass, pH, dissolved oxygen, and fluorescence. High oxygen transfer efficiency allows to carry out microbial culture in fully aerobic conditions and fed-batch and pH control are available, ensuring the compatibility of the results with those gained in conventional stirred bioreactor. Fluorescence sensor available in each well can be used to gain informations at the level of a fluorescent reporter system, but only at the bulk level. Other mini-bioreactor systems are now available; either based on the concept of “shaken” bioreactor (e.g., Micro 24-microreactor system developed by Pall) or “stirred” bioreactor (e.g., the 48-bioreactor system developed by 2mag) (Lattermann and Buchs, 2014). Single-cell results can be obtained by coupling the cultivation device to a robotic platform delivering the samples to a flow cytometer. Microbial phenotypic heterogeneity is a phenomenon that has gained a lot of attention, considering its potential impact on bioprocesses (Delvigne et al., 2014). Fluorescent reporter library is a technology of choice for investigating the effect of microbial phenotypic heterogeneity on bioprocesses and single-cell analytical devices compatible with bioreactor are needed at this level. Indeed, genome-scale investigation of GFP reporter libraries have led to a better understanding of the evolution of noise in gene and protein expression (Taniguchi et al., 2010; Silander et al., 2012) and the associated molecular mechanisms (Swain et al., 2002). Since on-line flow cytometry can be adapted to monitor phenotypic heterogeneity during a microbial culture by coupling a specific interface to the cultivation device (Brognaux et al., 2013), these mechanisms are now considered in bioprocessing conditions (Polizzi and Kontoravdi, 2014; Baert et al., 2015). Several interfaces have been built for this purpose and have been successfully used to monitor the activity of fluorescent reporter in bioprocess conditions (Abu-Absi et al., 2003; Arnoldini et al., 2013; Besmer et al., 2014; Delvigne et al., 2015). In the following section, the use of fluorescent reporter libraries will be illustrated for different biotechnological applications.

## Biotechnological Applications Related to the Use of Fluorescent Reporter Systems

### Production of recombinant proteins: From promoter strength to noise in expression

The use of transcriptional reporter, i.e., a promoter linked to the sequence of a fluorescent protein, allows for the fast and non-invasive characterization of the strength of an expression vector and the protein synthesis rate over time (DeLisa, 1999; Zaslaver et al., 2004). This type of information is very useful when optimizing a process and is greatly facilitated by the fluorescent signal that can be detected on-line (see Figure 1 for an overview of the combination between cultivation devices and recording tools available). Translational reporters, i.e., a promoter linked to the sequence of a protein of interest either homologous or heterologous, this latter being tagged with a fluorescent protein facilitating the detection of the whole chimeric protein, can be used for the optimization of the production of a recombinant protein, i.e., by adjusting the induction profile if the promoter is inducible or more generally by adjusting the cultivation conditions (DeLisa, 1999; Abu-Absi et al., 2003). However, several factors must be taken into account for the design of an efficient translational reporter. Indeed, using a fluorescent protein as a tag can have a profound impact on protein folding and stability, and specific technologies, such as split-GFP, have been designed for this purpose (Cabantous et al., 2005). Another drawback is that the signal gained from a fluorescent reporter accounts only for a product accumulated intracellular. It is thus theoretically not possible to get informations about the amount of products secreted, which is an important limitation since most of the biotechnological applications are oriented toward product secretion to the extracellular medium in order to decrease the costs associated with downstream processing operations. In this context, a specific micro-cultivation device based on micro-engraving has been developed and allows not only for the analysis of the intracellular level of fluorescent protein but also for the amount of fluorescent protein excreted at the extracellular level (Love, 2010, 2012). Another strategy for the detection of excreted compounds include the use of surface display or droplet microfluidics (Hai and Magdassi, 2004; Kintses et al., 2012; Mazutis et al., 2013).

### Detection of metabolite productivity at the intracellular level

Besides the use of promoter-based fluorescent reporter, other biosensing strategies can be designed for the detection of intracellular metabolites. The most widely used strategy for the detection of intracellular metabolites relies on the use of transcriptional factors (Eggeling et al., 2015). In this context, the interaction of the metabolite with a transcriptional factor induces the synthesis of the fluorescent reporter molecule. Another strategy relies on the use of riboswitches where the binding of the metabolite to a RNA aptamer triggers the synthesis of the fluorescent reporter molecule. However, this strategy is less used than the design based on transcriptional factors. This kind of sensing strategy is invaluable for the design of hyper productive strains (Eggeling et al., 2015). For example, Binder and co-workers used transcription factor-based biosensor for the selection of l-lysine hyperproducers among a population of *Corynebacterium glutamicum* exposed to chemical mutagenesis (Binder, 2012, 2013). The fluorescent biosensor facilitated the high-throughput detection by flow cytometry, as well as the isolation of the mutants by fluorescence-activated cell sorting (FACS). A similar workflow has also been applied to other relevant cell factories, such as *S. cerevisiae* and *E. coli* (Delvigne and Goffin, 2014).

### Synthetic biology and the optimization of artificial metabolic pathways

Synthetic biology allows for more important modification of cell factories, such as the integration of artificial metabolic pathways on specific microbial chassis (Silva-Rocha and de Lorenzo, 2010; Martinez-Garcia et al., 2014). However, synthetic biology requires the use of reliable parts for the design of effective cell factories. Such parts can now be found in dedicated libraries, such as those of the BioBrick initiative (Rokke et al., 2014). However, the biobrick registry is based mainly on standardized parts designed for easily assembling synthetic vectors. Since this initiative, other registries have been designed on the basis of more functional parts. As an example, the ePathBrick library can be used to insert artificial metabolic pathways inside *E. coli* for the synthesis of diverse chemicals (Xu et al., 2012). Such libraries and assembly protocols allow for the fast optimization of multi-gene pathways with different gene configurations inside a specific host (Jones et al., 2015). One of the requirements of synthetic assembly is the orthogonality, i.e., the lack of interferences coming from biochemical interactions between artificial elements (Michener et al., 2012). In this context, fluorescent reporter system can be used for the fine-tuning of the different gene expression systems required for the artificial pathway (Schendzielorz, 2013; Nikel et al., 2014a). This helps finding the best vector configurations allowing the balanced expression of all the enzymes involved in the artificial pathway.

### Design of complex phenotypes for food and white biotechnology applications: Directed and laboratory evolution

All of the above-mentioned examples imply the use of genetically modified strains. However, there are a lot of biotechnological applications where GMOs are not allowed, such as for agro-food applications for the development of starters and probiotics. More generally, there are a lot of biotechnological applications where complex phenotypes, combining robustness and productivity of the microbial cell factory, are needed. Such phenotypes are needed for the development of white biotechnology applications, i.e., the production of fuels and chemicals from complex lignocellulosic substrates containing inhibitors (Vasdekis and Stephanopoulos, 2015). In this context, the fluorescent reporter technology would give also invaluable insight about the phenotypic differentiation leading to the appearance of such phenotypes. Indeed, fitness is linked with noise in biochemical processes, and noise can be studied by the use of specific fluorescent reporter (Abee et al., 2011; Ryall et al., 2012; Holland et al., 2013). These reporters can be used to study bet hedging, a phenomenon by which a microbial population exploit phenotypic noise in order to increase its fitness in an ecosystem (Veening, 2008). Bet hedging has been recently identified as a major mechanism in diauxic shift (Boulineau et al., 2013; Solopova et al., 2014; van Heerden, 2014). Since diauxic shift arises frequently in bioprocesses, and especially within those based on complex substrate, it is of importance to control this mechanism. On the other hand, the design of complex phenotypes can be obtained by directed or natural evolution of a microbial strain (Dragosits and Mattanovich, 2013). At this level, also fluorescent reporters can be used to visualize in real time laboratory evolution (VERT). The VERT system is based on competitive fitness principle with different strains placed simultaneously in the cultivation system (Reyes, 2012a,b). The best strain can be easily selected since each strain carries a fluorescent reporter exhibiting a specific color. This technique allows for the fast detection of evolved phenotypes, the reporter plasmid being cured at the end of the experiments in order to meet the non-GMO requirements.

## Drawbacks Associated with Promoter-Based Fluorescent Biosensors

**Figure 2:**
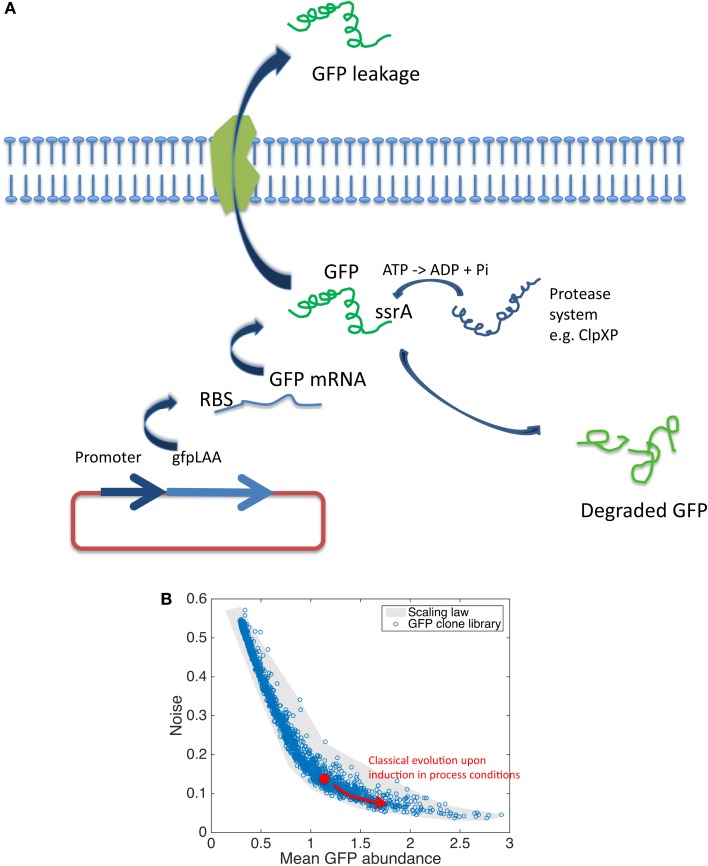
**(A)** Scheme showing the different molecular mechanisms affecting the intracellular content in GFP. **(B)** Scaling law adapted from Silander et al. (2012) showing the relationship between the level of promoter activity (in log scale) and its cell-to-cell variability or noise (the noise is calculated as the ratio between the standard deviation and the mean GFP content among a population of microbial cell, in log scale). The red arrow shows a typical evolution in GFP expression in real bioprocessing conditions (Baert et al., 2015).

Reporter-based fluorescent biosensors have been widely used in the context of bioprocess optimization (Polizzi and Kontoravdi, 2014). However, some precautions must be taken since several factors can have a significant impact on the synthesis of the reporter molecules. These factors are summarized at Figure 2A and comprises the plasmid copy number, the strength of ribosome binding site (RBS), the folding rate of the GFP and its stability, and the potential release of GFP to the extracellular medium. Plasmid copy number is known to affect the degree of expression of GFP since this number can vary during cultures according to many environmental and intrinsic conditions. However, it has been shown that this effect can be limited by using low-copy number plasmids or by considering chromosomal integration (Freed et al., 2008; Silander et al., 2012). The strength of the RBS can also affect the rate by which mRNA is translated into GFP and this factor is now integrated when designing synthetic gene circuits (Xu et al., 2012; Jones et al., 2015). Typical folding rate of engineered GFP (e.g., GFPmut2 and GFPmut3) are below 4 min (Cormack, 1996), but the stability is very high (>24 h) leading to cumulative signal. Destabilized version of GFP have been designed and used to monitor gene expression in bioprocessing conditions (Han et al., 2013; Hentschel et al., 2013). Destabilization of GFP relies on the use of specific *ssrA* tags that can be recognized by the internal protease machinery (i.e., *ClpXP* mainly). By varying the sequence of the last three amino acids, the affinity of the proteases can be modulated, leading to medium (e.g., GFPAAV for *E. coli* with a half-life of 40 min) to highly destabilized variants (e.g., GFPLAA for *E. coli* with a half-life of 10 min). However, in this case, the GFP response is affected not only by the real promoter activity but also by the content of intracellular protease and the adenosine triphosphate (ATP) availability since *ClpXP* is ATP dependent (Purcell et al., 2012; Han et al., 2013). These side-reactions can notably artificially enhance the cell-to-cell variability at the level of the GFP content (Baert et al., 2015). A last effect potentially affecting the overall level of GFP is its potential release to the extracellular medium. Indeed, increase in membrane permeability and protein leakage is known to occur when microbial cells are exposed to nutrient limitation, a condition encountered in fed-batch bioprocesses (Shokri, 2002, 2004). At this level, it has been shown that GFP can be subjected to such release (Delvigne et al., 2011; Brognaux et al., 2014). In the case of promoter-dependent reporter systems, all these side-reactions affect the reliability of the fluorescent signal according to the real activity of the targeted promoter. Also, the impact of cultivation conditions can also have a significant impact both on the mean expression level of GFP and on its cell-to-cell variability (called noise). Noise in GFP expression is indeed a very important factor that can have detrimental effect on productivity (Delvigne and Goffin, 2014; Delvigne et al., 2014). However, noise in gene expression is also recognized as a beneficial factor in the context of microbial robustness. Indeed, cell-to-cell heterogeneity increases the potential resistance of the microbial population upon stress exposure by a mechanism named bet-hedging (Holland et al., 2013; Nikel et al., 2014b; Martins and Locke, 2015). Since productivity and robustness are two traits that are generally expected from an efficient cell factory, the effect of noise have to be balanced between these two phenotypic traits and advanced analytical devices are awaited at this level (see Figure 1 for a description of such devices). At this level, the use of an *E. coli* GFP clone library (Zaslaver et al., 2006) in connection with high-throughput flow cytometry has allowed for the design of a scaling law (Figure 2B). The collection comprised more than 1500 promoter-based biosensors and has been used for a genome-wide screen of the relationship between the promoter activity and the noise (Silander et al., 2012). The scaling-law shows a clear relationship between the mean promoter activity and the noise in GFP expression, i.e., promoters with high expression level exhibiting a low amount of noise, whereas weak promoters tend to exhibit larger level of noise (Figure 2B). More recently, this scaling-law has been validated by on-line flow cytometry in real bioprocessing conditions (Baert et al., 2015). This scaling-law constitutes an invaluable tool for a better understanding of the expression efficiency of microbial systems. The study of microbial physiology based on the use of fluorescent reporter system is thus critical in order to increase our knowledge for the development of more efficient microbial cell factories. However, this development is slowed down by the development of cultivation devices compatible with single-cell analysis and by the need for reliable fluorescent reporter systems (Polizzi and Kontoravdi, 2014). Indeed, we have shown that promoter-based fluorescent reporters are subject to many side-reactions affecting the interpretation of the fluorescent signal. Efforts must thus be given at the level of the development of more reliable expression systems. Promoter-independent fluorescent reporter systems described in the Section “Detection of Metabolite Productivity at the Intracellular Level” and the developments of synthetic devices described in the Section “Synthetic Biology and the Optimization of Artificial Metabolic Pathways” are very promising at this level.

## Promises of Promoter-Independent Fluorescent Biosensors

According to previously established classifications, three types of fluorescent biosensors can be distinguished, i.e., promoter-based, transcriptional regulator, and RNA switches (Eggeling et al., 2015; Zhang et al., 2015). Besides these three main classes, a fourth one can also be considered, i.e., fluorescent protein based biosensors (Delvigne and Goffin, 2014; Liu et al., 2015). This type of biosensor relies on the constitutive expression of fluorescent protein linked with metabolite-binding domain and can be used for the intracellular detection of small molecules, such as ATP, cyclic adenosine mono-phosphate (cAMP), sugars, and organic acids, among others. Adequate constitutive expression of the fluorescent protein allows to get rid of most of the drawbacks depicted at Figure 2A and leads to a very dynamics signal that can be easily recorded by standard techniques (Figure 1). Additional advantages of this simple technology are the direct detection of important intracellular metabolites and its applicability to a broad range of hosts (Delvigne and Goffin, 2014). RNA switches, or riboswitches, can also be applied in the same context. These biosensors are based on mRNA elements able to sense metabolite concentration and modulate transcription and translation of a fluorescent protein accordingly. Their main drawback is the limited number of applications available so far (Eggeling et al., 2015), partially attributed to the fact that only a few number of natural mRNA regions with specific ligand properties are available. However, synthetic biology approaches allow for the design of artificial riboswitches with ligand capabilities for virtually all possible metabolites to be detected (Pothoulakis et al., 2014; Jang et al., 2015; You et al., 2015). Transcription-based fluorescent biosensors are based on the natural capabilities for transcription factor to sense protein interaction or change in intracellular metabolite concentration and regulate gene expression accordingly. This property can be used in order to drive the expression of a fluorescent protein in front of specific stimuli. This class of biosensor has been thoroughly used for monitoring the concentration of intracellular compounds (i.e., metabolites, co-factors, etc.) in metabolic engineering studies. However, since it relies on the synthesis of fluorescent proteins, it suffers from the same molecular drawbacks than classical promoter-based biosensors (Figure 2A). Another drawback is its host specificity. However, synthetic biology has been recently used for the design of more robust and widely applicable transcriptional biosensors (Zhang et al., 2015).

## Conclusion and Future Perspectives

The different examples shown in the previous sections point out that fluorescent reporter libraries have become a useful experimental tool used in different contexts for the optimization of the microbial cell factories, not only in the field of recombinant products but also for the production of metabolites, i.e., for detection of highly productive phenotypes and the orientation of the synthetic biology approaches.

In addition, the panel of experimental devices available for the analysis of such library is very large (Figure 1) and appropriate combination of such device on the basis of fluorescent reporter libraries allows for the high-throughout development of cell factories, from the selection of the best producers to the optimization of large-scale processes. However, additional efforts must be given in order to better integrate phenotypic noise. Indeed, cell-to-cell variation is an important factor known to affect bioprocess productivity and a specific mathematical background have to be set up in order to characterize this important phenomenon.

We have shown that most of the studies published actually are based on promoter-dependent fluorescent reporters. These molecular devices exhibit a lot of artifact that can lead to strong variation in fluorescent molecules expression. At this level, the development of promoter-independent fluorescent reporter would lead to more reliable clone libraries that can be used for the fast optimization of microbial cell factories.

## Conflict of Interest Statement

The authors declare that the research was conducted in the absence of any commercial or financial relationships that could be construed as a potential conflict of interest.
